# Small but big player: the important role of microRNAs in legume crops

**DOI:** 10.1007/s00438-026-02378-3

**Published:** 2026-03-11

**Authors:** Flavia Thiebaut, Maria Clara Urquiaga, Paula Machado de Araújo, Aislan de Carvalho Vivarini, Clicia Grativol

**Affiliations:** 1 Laboratório de Bioquímica e Biologia Molecular de Plantas e Microrganismos, Departamento de Biologia Celular e Molecular, Instituto de Biologia, Universidade Federal Fluminense, Bloco M - Rua Prof. Marcos Waldemar de Freitas Reis - São Domingos, , Niterói, RJ 24210-201 Brazil; 2 Laboratório de Biologia Molecular de Plantas, Instituto de Bioquímica Médica Leopoldo de Meis, Universidade Federal do Rio de Janeiro, Cidade Universitária, Avenida Carlos Chagas Filho, 373, CCS, Bl.L-29ss, Rio de Janeiro, RJ 21941-599 Brazil; 3 Laboratório de Química e Função de Proteínas e Peptídeos, Centro de Biociências e Biotecnologia, Universidade Estadual do Norte Fluminense Darcy Ribeiro, Avenida Alberto Lamego, 2000, P5 – sala 228, Parque Califórnia, Campos dos Goytacazes, RJ 28013- 602 Brazil; 4 Laboratório de Biologia Molecular Mitocondrial, Departamento de Biologia Celular e Molecular, Instituto de Biologia, Universidade Federal Fluminense, Bloco M - Rua Prof. Marcos Waldemar de Freitas Reis - São Domingos, Niterói, RJ 24210-201 Brazil

**Keywords:** Small RNA, Plant development, Gene regulation, Environmental response, Biotechnological application

## Abstract

Legumes are essential components of global cropping systems due to their nutritional value and contribution to sustainable agriculture. Among the regulatory molecules, small RNAs (sRNAs), particularly microRNAs (miRNAs), play crucial roles in plant development and in responses to biotic and abiotic stresses. miRNAs regulate genes involved in diverse developmental processes, including nodule formation, which is fundamental for the nitrogen-fixing symbiosis that characterizes legumes. Functional studies have demonstrated that miRNAs are key modulators of plant defense, contributing to resistance against pathogens and environmental challenges. Moreover, miRNAs also participate in cross-kingdom communication, such as plant–bacteria interactions, influencing symbiotic efficiency. Advances in molecular biology have enabled the manipulation of miRNAs and their targets for crop improvement. Current approaches include the design of artificial miRNAs (amiRNA), modulation of miRNA expression through miRNA-encoded peptides, genome editing of non-coding genes using CRISPR/Cas9, and the application of RNA interference (RNAi) technology. Together, these strategies highlight the potential of miRNA-based tools in plant biotechnology. A deeper understanding of the molecular mechanisms governing miRNA-mediated gene silencing will provide powerful resources for optimizing legume productivity and resilience within sustainable agricultural systems.

## Introduction

Legumes are a major component of all cropping system worldwide and commonly recognized for their nutritional benefits and influence in the sustainability of agricultural systems. Legume crops, including species such as soybean, chickpea, and common bean, are fundamental to global agriculture, supplying essential nutrients for human and animal consumption and enhancing soil fertility through biological nitrogen fixation (Foyer et al. 2016; Maphosa And Jideani 2017; Meena And Lal 2018). The latter is due to the fact that most legumes establish a mutualistic relationship with nitrogen-fixing rhizobia. This interaction causes legumes to provide around 21 Mt of nitrogen for agriculture on a global scale (Foyer et al. 2016), making these species essential crops for sustainable agricultural production.

The success and productivity of these crops are intricately tied to their genetic makeup and regulatory mechanisms, which govern crucial developmental processes, stress responses, and symbiotic interactions (Jha et al. 2021). However, the constant challenges due the global climate changes emphasizes the need for efficient research approaches in order to develop crops capable of tolerating environmental variations, increasing productivity and quality (Kope 2024). Several researches have contributed to elucidating physiological and molecular components subjacent stress responses of a wide diversity of legume species (Deshmukh et al. 2014; Taïbi et al. 2016; Matamoros And Becana 2021; Singer et al. 2023).

In the dynamic of plant molecular biology, small RNA molecules (sRNA) have emerged as central players in the intricate regulatory networks governing gene expression and phenotype (Khan et al. 2011; Formey et al. 2015; Sun et al. 2016; Jha et al. 2021; Castaingts et al. 2022). Among these sRNA species, microRNAs (miRNAs), with a size of approximately 20–24 nucleotides (nt), have garnered considerable attention for their ability to fine-tune gene expression and mediate adaptive responses to environmental cues (Phillips et al. 2007). Moreover, emerging evidence suggests that miRNAs play a central role in mediating symbiotic interactions between legumes and nitrogen-fixing bacteria, such as rhizobia, thereby influencing nodulation and nitrogen fixation efficiency (Yan et al. 2013; Hoang et al. 2020). While many miRNAs are evolutionarily conserved and regulate fundamental processes across plant species, non-conserved miRNAs, often lineage- or species-specific, play important roles in species-specific cellular processes (Hernández et al. 2025). For instance, miR1509 is a legume-specific miRNA demonstrated to control nodulation in chickpea (Tiwari et al. 2021).

Despite some specific features, conserved and non-conserved miRNAs are produced through a highly regulated canonical biogenesis pathway (Hernández et al. 2025). miRNA biogenesis begins in the nucleus with the transcription of MIR genes by RNA Polymerase II (Pol II), in association with the NOT2 protein and the MEDIATOR complex (Kim et al. 2011; Wang et al. 2013). MIR genes are predominantly intergenic, although some originate from introns or other non-coding RNAs (Knop et al. 2017). The primary transcript (pri-miRNA) forms an imperfect hairpin structure and contains a 5′ cap and a 3′ poly-A tail (Lee et al. 2004). Pri-miRNAs are processed by Dicer-like 1 (DCL1) within the Microprocessor complex, together with HYL1 and SE, generating a ~ 21-nt miRNA/miRNA* duplex (Kurihara et al. 2006). This duplex is stabilized by 3′-end methylation mediated by HEN1 (Yu et al. 2005). Although initially considered non-functional, the miRNA* strand has been shown to participate in gene regulation in soybean (Luo et al. 2012; Liu et al. 2017). The duplex is exported to the cytoplasm mainly by the Exportin 5 homolog HASTY (HST), although RISC loading may also occur in the nucleus prior to export via Exportin 1 (Bologna et al. 2018; Park et al. 2005). In the cytoplasm, the mature miRNA is incorporated into the RNA-induced silencing complex (RISC), with ARGONAUTE 1 (AGO1) as its core component (Baumberger And Baulcombe 2005). In legumes, miRNAs predominantly regulate gene expression through target mRNA cleavage mediated by near-perfect base pairing, although translational repression and epigenetic regulation have also been reported (Chen 2004; Kidner And Martienssen 2005; Wu et al. 2010).

**Fig. 1:**
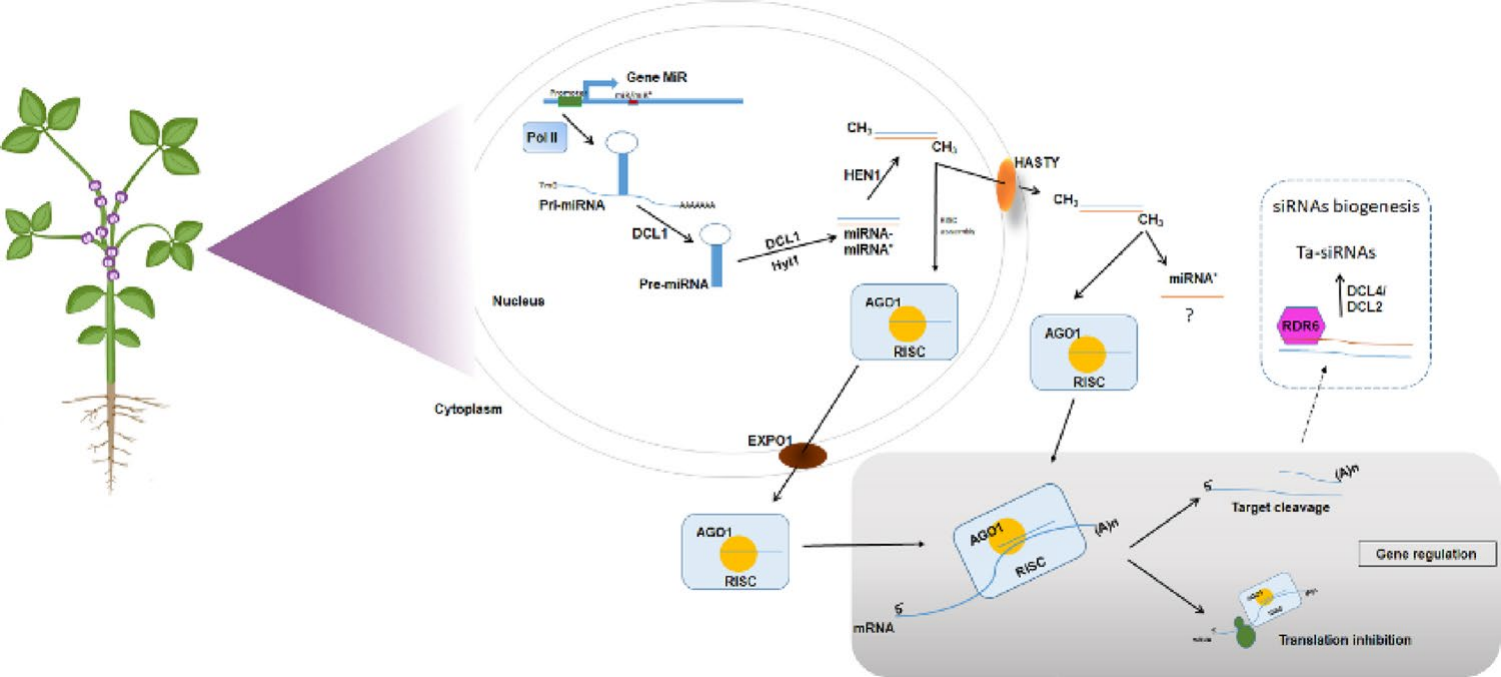
Simplified schematic model of miRNA biogenesis and action in plants. First, *MIR* genes are transcribed by RNA Polymerase II (Pol II) into the primary miRNA (pri-miRNA). This precursor having a 7 ‘methyl guanosine cap at the 5’ end and a poly-A tail at the 3 ‘end and is mainly processed by DCL1. DCL1 interacts with HYPONASTIC LEAVES 1 (HYL1) and leads to the formation of the miRNA/miRNA∗ duplex. HEN1 methylates the duplex and it can be transported to the cytoplasm by HASTY. RISC assembly can be occurring in the cytoplasm, or can also occur in the nucleus, being transported by EXPO1. The possible mechanisms of action of miRNAs are in the gray box. Additional action of miRNA is trigger the production of ta-siRNAs through siRNA biogenesis via mechanisms involving RDR6 and DCL2/4

The strong evolutionary conservation of core miRNA biogenesis components across land plants highlights the fundamental role of this regulatory pathway (Bajczyk et al. 2023). Figure 1 summarizes the main steps of miRNA biogenesis and mechanisms of gene regulation.

Building on this conceptual framework, the subsequent sections examine how these regulatory mechanisms operate in legumes, highlighting the functional significance of specific miRNAs in developmental processes and adaptive responses to environmental stimuli. First, we describe the miRNAs implicated in legume development, followed by those associated with diverse environmental stimuli, including abiotic and biotic stresses as well as plant–rhizobia interactions. Finally, we discuss the emerging prospects for the biotechnological application of miRNAs in legumes. By elucidating the complex interplay among miRNAs, gene regulation, and environmental cues, this work establishes a conceptual foundation for future research aimed at leveraging sRNA pathways to enhance crop performance and promote sustainable agriculture.

## The regulatory function of miRNAs in legume development

**Fig. 2:**
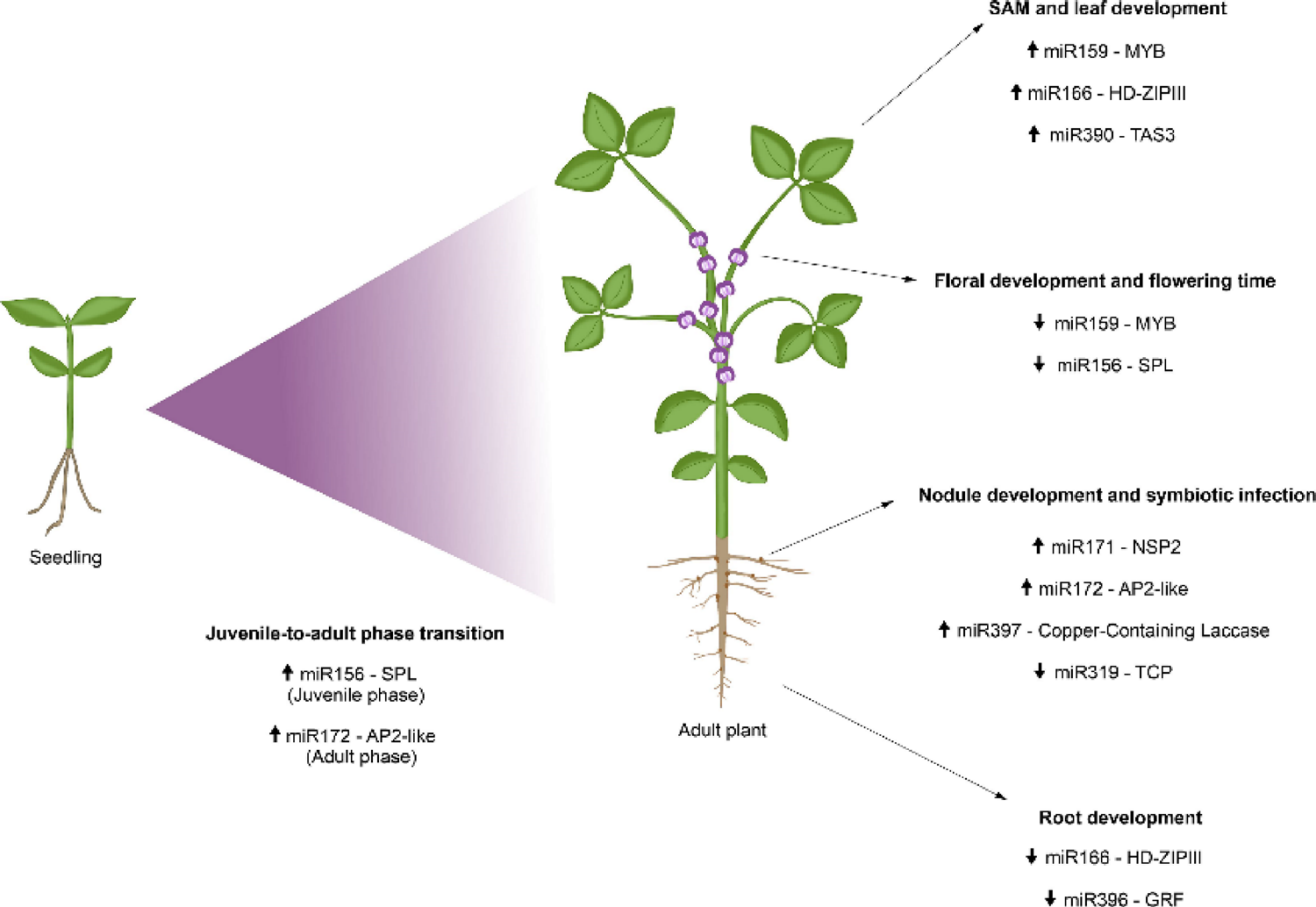
miRNAs involved in different stages of development in legumes and their respective targets. Arrows represent miRNA expression during legumes development. up arrow: up-regulation; down arrow: down-regulated

Dysregulation of miRNA biogenesis in plants results in severe developmental defects, and compromises, for example, plant size, root and floral organ development, and leaf shape (Li and Zhang 2016). In *Glycine max* (soybean), mutants of *DCL1* homologs, *DCL1a* and *DCL1b*, required for miRNAs biogenesis, showed developmental defects, including reduced seed size and interrupted seedling development, and also presented affected miRNA accumulation and target gene expression (Curtin et al. 2016). In *A. thaliana*, the *dcl1* mutants exhibit alterations in ovule, embryo, and seedling development, which demonstrates the fundamental role of miRNAs since the early stages of plant development (Schauer et al. 2002). Moreover, in soybean, *DCL2* inactivation affects the biogenesis of small RNAs, with reduced number of 22-nt sRNAs in *Gmdcl2a* and *Gmdcl2b* mutants, affecting the seed coat color (Jia et al. 2020). Figure 2 shows some of the main miRNAs related to legumes development.

In legumes, most of the miRNAs involved in developmental processes have been reported in *Medicago truncatula* and soybean. The evolutionarily conserved miRNAs miR156 and miR172 play a regulatory role during the juvenile-to-adult phase transition, targeting *SQUAMOSA PROMOTER BINDING PROTEIN-LIKE* (*SPL*) genes and *APETALA2-like* (*AP2-like*) genes, respectively (Huijser And Schmid 2011). Another study demonstrated that the expression levels of miR156 and miR172 in soybean are equivalent to what has already been reported for Arabidopsis, maize, and rice (Yoshikawa et al. 2013). These miRNAs exhibit inverse expression patterns. MiR156 has high expression in the juvenile phase and its level decreases as the plant develops. Conversely, miR172 shows low expression in the juvenile phase and its level increases in the adult phase. The coordinated regulation made by miR156 and miR172 is essential for the correct transition from the vegetative phase, being a common regulatory mechanism among different plant species (Yoshikawa et al. 2013).

Different miRNAs act during the development of the shoot apical meristem and leaves. MiR159, miR166, and miR390 are expressed in apical shoot meristem and leaves in soybean, which are important for leaf development (Wong et al. 2011). The members of the miR159 family are conserved in most land plants with various functions in vegetative tissues via regulation of *MYB* genes (Millar et al. 2019), while miR166 targets the *Homeodomain-leucine zipper III* (*HD-ZIPIII*) genes and is involved in leaf polarity establishing (Chitwood et al. 2007). MiR390 regulates *TAS3* transcripts, which trigger the tasiRNAs production, involved in the regulatory network of *Auxin response factor* (*ARF*) genes (De Felippes et al. 2017). An interesting regulation of adaxial–abaxial leaf polarity in maize was demonstrated through the coordinated action of specific small-interfering RNAs (tasiR-ARF) and miR166 (Nogueira et al. 2007). While the accumulation of tasiR-ARF directs the establishment of the adaxial domain, miR166 determines the abaxial domain, via regulation of *HD-ZIPIII* genes. In soybean, the miR390 is expressed on the adaxial side of the emerging leaf primordia, and miR166 is present on the abaxial side of the incipient leaf (Wong et al. 2011), which corresponds to the pattern reported in maize (Nogueira et al. 2007). In addition, the expression pattern observed in older leaf primordia suggests that miR390 can also regulate leaf vein formation in soybean (Wong et al. 2011).

Besides acting on leaf development, miR166 is also related to roots and nodules formation in *M. truncatula*, regulating *HD-ZIPIII* genes. MiR166 overexpression and consequent downregulation of *HD-ZIPIII* genes causes the reduction of lateral roots and nodules, and affects the development of vascular bundles (Boualem et al. 2008). miR396 is also involved in legume root development, targeting *Growth-regulating factors* (GRFs). In *M. truncatula*, when miR396 is overexpressed or inactivated, the expression of *GRF* genes is affected, interfering with root growth and colonization by mycorrhizal fungi. Furthermore, the number of dividing cells, as well as the expression of cell-cycle genes are altered, which reinforces the importance of miR396 in the root meristem activity (Bazin et al. 2013). In soybean, miR396 expression was identified in young roots. It was also shown that the regulatory network between miR396 and *GRFs* is essential for correct root formation in soybean (Noon et al. 2019).

miR172 has a vital function in nodule development in legumes, through the regulation of *Apetala2* transcription factor (Yan et al. 2013). This miRNA was reported in the legume species *Phaseolus vulgaris* (common bean), *Lotus japonicus*, *M. truncatula*, and soybean (Lelandais-Brière et al. 2009; De Luis et al. 2012; Wang et al. 2014b; Nova-Franco et al. 2015). In soybean, miR172c can regulate rhizobial infection and nodule organogenesis (Wang et al. 2014b). In common bean, increased expression of miR172c is observed after rhizobia infection, during nodulation, and in mature nodules. Plants overexpressing miR172c show enhanced nodulation and nitrogen fixation, increased rhizobial infection and improved root growth (Nova-Franco et al. 2015).

In *L. japonicus*, two other miRNAs have been reported, miR171 and miR397, which are related to symbiotic infection and nodule function. miR171 targets the nodulation transcription factor *Nodulation Signaling Pathway 2* (*NSP2*) and shows stable expression levels during nodule developmental stages. The analyzed isoform miR171c is up-regulated in infected nodules, but it shows low expression in uninfected nodules, suggesting the response of this miRNA to symbiotic infection. MiR397 regulates a *Copper-Containing Laccase* gene and is associated with nitrogen fixation-related copper homeostasis. In contrast to miR171c, miR397 shows high expression specifically in mature nodules, and possibly acts on nodule senescence and nodule functionality (De Luis et al. 2012). In *M. truncatula*, overexpression of a miR319 isoform was shown to reduce the expression levels of its *Teosinte branched1*, *Cycloidea*, and *Proliferating cell nuclear antigen binding factor* (*TCP*) targets, resulting in decreased nodule number and weight (Wang et al. 2018). This finding indicates that the *TCPs* regulation by miR319 can modulate the development of nodules. In *P. vulgaris* nodules, an increased expression of miR319d and a decline in *TCP* targets were identified, which suggests that miR319 also acts in the development of nodules of this legume (Formey et al. 2015).

Another important developmental stage in which miRNAs operate in plants is the floral development. One of the main miRNAs that mediate flower development is miR159 (Waheed and Zeng 2020). This miRNA regulates *GAMYB*-related genes, involved in the activation of the *Leafy* gene, and is fundamental for proper anther development (Achard et al. 2004). In soybean, the miR159 accumulates in petal, carpel, and stamen tissues. The increased expression of miR159 leads to a decrease in Leafy transcript levels, affects anther development and changes flowering time, coinciding with the study carried out in Arabidopsis (Achard et al. 2004; Kulcheski et al. 2016). The flowering time can also be modulated by miR156-SPLs regulation. Overexpression of miR156 causes delay in flowering time, which has been observed in different species, including soybean and *Medicago sativa* legumes (Wang et al. 2014a; Cao et al. 2015; Gao et al. 2018). In addition, in the soybean cytoplasmic male sterility (CMS) line, miR156b regulates floral organ development by regulating GmSPL and downstream genes such as *Lateral organ boundaries domain* and *Agamous-like*, involved in pollen development and floral organ differentiation, respectively (Ding et al. 2020). In yellow lupine (*Lupinus luteus*), miR156 and miR159 represent two of the most abundant miRNA families, suggesting their significant regulatory roles in flower development (Glazińska et al. 2019).

miRNAs also play an important role in legume seed development. In soybean, for example, miR396 functions as a negative regulator of seed size and yield by repressing *GRF* genes. Knockout mutants of multiple miR396 family members produced larger seeds and, in some cases, increased yield, making them valuable targets for molecular breeding of high-yield varieties (Xie et al. 2024). In chickpea, miR164e negatively regulates seed protein accumulation by directly targeting the transcription factor NAC100, which activates seed storage protein (SSP) genes. Downregulation of miR164e during seed maturation allows NAC100 to promote protein-rich seeds, while its overexpression suppresses protein content (Chakraborty et al. 2024).

The results described here confirm that many miRNAs are involved in legume development. These miRNAs are differentially regulated depending on the plant growth phase, for example, miR156 is induced in the juvenile phase, while miR172 is induced in the adult phase of the plant. Furthermore, several miRNAs have been described as important in the development of leaves, flowers, seeds, and roots and even in the formation of nodules.

## Deciphering the regulatory role of miRNAs in legume-environment interactions: insights and implications

**Fig. 3:**
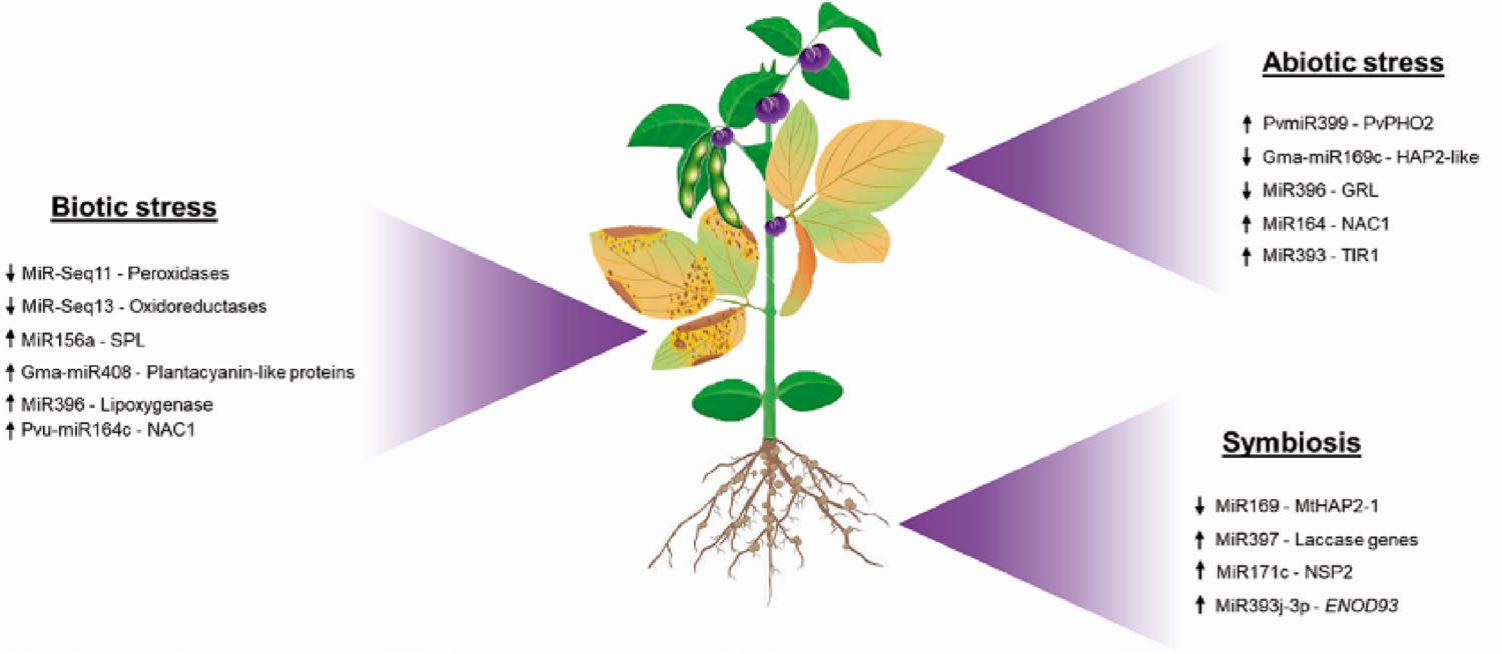
Schematic representation of legume miRNAs regulated in response to different environmental stimulus: Biotic stress, Abiotic stress and Symbiosis. Arrows represent miRNA expression in legumes submitted to environmental stimulus. up arrow: up-regulation; down arrow: down-regulated

Plants have developed sophisticated mechanisms to tolerate environmental stresses by modulating the expression of a varied set of genes. The great ability of miRNAs to act as efficient regulatory elements both in response to stress conditions and in plant development resulted in a paradigm change in the knowledge of post-transcriptional gene regulation (Singh et al. 2017). These molecules have been described as essential for plant development, reproduction, and genome reprogramming, regulating the expression of their targets in response to different stimuli and contributing to plant phenotypic plasticity. Figure 3 shows some miRNAs identified in legumes as regulated in response to different environmental stimulus.

### MiRNA regulation in legume responses to abiotic stress

Abiotic stresses are considered the main challenges to global food security, since they negatively impact crop productivity as they represent more than 50% of crop production losses worldwide (Oshunsanya et al. 2019). Besides that, they can influence grain composition and can inhibit nodule development (Valentine et al. 2010; Farooq et al. 2018; Sarkar et al. 2021). Like other crops, legumes productivity is significantly affected by several environmental stresses such as salinity, temperature, drought and nutrient deficit (Signorelli et al. 2015; Jimenez-Lopez et al. 2023). Many studies have identified legumes miRNAs regulated in response to stress conditions, and some have even been able to predict their possible targets, unraveling an important functional role of these miRNAs (Naya et al. 2014; Li et al. 2017b). The first abiotic stress-responsive miRNA described in legumes was identified in common bean (*Phaseolus vulgaris*) under phosphorus deficiency (Valdés-López et al. 2008).PvmiR399 plays a central role in phosphate starvation signaling, contributing to the regulation of phosphate homeostasis, transport, and adaptive nutrient responses.

Although legumes naturally encounter fluctuating temperatures (Jyothi et al. 2015; Bhandari et al. 2017), exposure to thermal extremes, both heat and cold, remains among the most deleterious stresses across developmental stages, ultimately curtailing physiological performance and translating into marked yield penalties (Kumar et al. 2010). Investigations into the regulation and regulatory roles of cold-responsive miRNAs in active nodules of soybean revealed that gma-miR169c is repressed under low-temperature treatment (Zhang et al. 2014). Rapid amplification of cDNA ends (5’-RACE) experiments suggests that gma-miR169c targets HAP2-like transcription factor. Similarly, *MtHAP2-1* encodes an NF-YA transcription factor targeted by miR169, in which case it is required for nodule development (Combier et al. 2006). Although direct miR169 targeting of other HAP2-like genes has not yet been demonstrated, it is plausible that these *HAP2*-like genes are miR169-responsive regulators of nodule cold responses (Zhang et al. 2014).

Interestingly, a recent study described that miR156, miR169 and miR5770 were downregulated in three different cultivars of soybean, showing clearly opposite expression patterns from that found in cold-sensitive variety (Kuczyński et al. 2022). Together with functional data of GO and KEGG annotations, the results indicate that these miRNAs may regulate the expression of genes related to plant abiotic stress response mechanisms such as reactive oxygen species (ROS) scavenging, flavonoid biosynthesis and regulation of osmotic potential (Kuczyński et al. 2022). Integrated sRNA and degradome sequencing were employed to assess miRNA functional roles in peanut (*Arachis hypogaea* L.) by comparing a cold-sensitive with a cold-tolerant variety (Zhang et al. 2022). The analysis revealed differential expressions of miRNAs (miR160, miR162, miR 396, miR482, miR1511 and miR2118) under cold stress, which likely contribute to tolerance via modulation of C-repeat binding factor (CBF) and auxin response factors (ARF) pathways, protein-kinase cascades, small-RNA biogenesis, and microtubule stability (Zhang et al. 2022). Using similar methodological approaches, miR4415, a legume-specific miRNA, was recently implicated in cold acclimation of *Ammopiptanthus nanus*, a rare temperate shrub (Zhu et al. 2022).Low temperature suppresses miR4415 expression, leading to the induction of its target, L-ascorbate oxidase (L-AO). Increased (L-AO) levels modify the apoplastic redox state, thereby contributing to freezing tolerance in seedlings (Zhu et al. 2022).

Salinity stress is one of the major environmental threats that significantly constraints crop production and consequently has negative effects on food security. Increased soil salinization can disturb overall legumes growth by influencing seed photosynthesis, germination and nutritional imbalance (Patil et al. 2016; Farooq et al. 2017; Khan et al. 2017). The functional roles of miRNAs in response to salt stress are not yet fully elucidated. Several studies have attempted to expand this knowledge in legumes; in *M. truncatula* (Long et al. 2015; Arshad et al. 2017; Cao et al. 2018; An et al. 2022); soybean (Dong et al. 2013; Wang et al. 2020a; Chen et al. 2024); *Vigna unguiculata* (Paul et al. 2011) and chickpea (*Cicer arietinum)* (Kohli et al. 2014; Khandal et al. 2017; Jatan et al. 2019).

In *Vicia faba*, exposure to salt stress (150 mM NaCl) resulted in the modulation of hundreds of miRNAs in two contrasting cultivars—284 induced and 243 repressed in the salt-sensitive cultivar, and 298 induced and 395 repressed in the salt-tolerant cultivar (Alzahrani et al. 2019). miR396 was repressed in both genotypes, consistent with findings in *Triticum dicoccoides*, where miR396 targets *Growth-Regulating Factor-like* (GRF) and heat-shock proteins linked to stress protection (Kantar et al. 2011). The role of miR396a in soybean development and salinity tolerance was assessed using overexpression and CRISPR/Cas9-edited lines (Chen et al. 2024). Based on MiR396a regulates growth-related traits through GRF transcription factors, edited lines displaying enhanced branching, seed yield, and salinity tolerance, while overexpression resulted in impaired development and reduced productivity (Chen et al. 2024). Another example is miR166m, which represents the first reported case of miRNA arm switching in soybean under salinity stress, with its two arms targeting distinct stress-related genes (Li et al. 2022). This arm-specific regulation highlights a flexible miRNA-mediated mechanism contributing to soybean responses to saline conditions. Water limiting in soils is considered the major environmental concern for agricultural systems (FAO 2017). Grain legumes depend on rainfall and are susceptible to water deficit during its vegetative and reproductive growth stages, severely reducing legume production worldwide (Daryanto et al. 2015). Such constraints reinforce the urgent need to clarify molecular mechanisms by which legumes respond to water deprivation. Drought-responsive miRNAs were identified in common bean (Arenas-Huertero et al. 2009; Sosa-Valencia et al. 2017; Wu et al. 2017); mungbean (Kumar et al. 2022b); grass pea (Bhat et al. 2020); soybean (Kulcheski et al. 2011; Zheng et al. 2016); cowpea (Mishra et al. 2022); peanut (Mittal et al. 2023); chickpea (Singh et al. 2025); alfalfa (Li et al. 2017b; Shi et al. 2021; Ruan et al. 2024; Wei et al. 2024); V. *unguiculata* (Barrera-Figueroa et al. 2011)d *sativa* (Zhu et al. 2021).

The function of miR408 under drought stress was examined in chickpea, the world’s second most widely cultivated legume, predominantly grown in arid and semi-arid regions (Hajyzadeh et al. 2015). Transgenic lines overexpressing the miR408 showed increased tolerance after 17 days of water deficiency. MiR408 overexpression resulted in the repression of plantacyanine transcripts expression, responsible for binding copper (Cu), causing Cu accumulation. Consequently, plantacyanin transcript repression caused regulation of drought-responsive genes, including dehydration-responsive element-binding protein (DREB) (Hajyzadeh et al. 2015). Additionally, DREB level was increased upon excess Cu (Ban et al. 2011). Similarly, an integrative sRNA sequencing and transcriptome study conducted on a drought-tolerant cowpea cultivar revealed the expression profile of drought-responsive miRNAs (Mishra et al. 2022). Among these, the up-regulation of miR408 in response to stress stood out. In this case, 5’-RACE experiments suggested *Laccase 12* (*LAC12*) as a potential target. Functional analysis using transgenic lines overexpressing the artificial miR408 showed greater tolerance to drought and salinity compared to wild-type plants (Mishra et al. 2022). *LAC12* expression, linked to lipid catabolism, was significantly reduced in drought-stressed wild-type and transgenic cowpea, suggesting that regulation of lignin content is a key trait underlying drought tolerance. Similar to observed in chickpea, another study showed that water deficit conditions led to increased accumulation of miR408 in *Medicago truncatula* (Trindade et al. 2010). In contrast, 23 miRNAs, including miR408, were downregulated under drought stress and subsequently upregulated during rehydration in *Medicago ruthenica* (Shi et al. 2021). These contrasting expression patterns likely reflect species-specific regulatory strategies and differences in drought intensity, duration, and experimental conditions. Despite their evolutionary conservation, miRNAs exhibit context-dependent regulatory divergence, resulting in distinct transcriptional responses under drought stress. Comparative analyses across species, coupled with functional validation under field-relevant conditions, will be essential to clarify the role of miR408 in drought adaptation. MiR166 is highly conserved in plants and is widely implicated in drought response and developmental pathways, particularly in the control of genes in the HD-ZIP-III family, transcription factors central to vascular tissue formation and leaf structure (Li et al. 2017a). Initial evidence from sRNA sequencing indicates that miR166 responds strongly to both exogenous nitric oxide and drought, connecting redox signals to water adaptation (Zhao et al. 2020b). In alfalfa, miR166 displays a dynamic drought-responsive regulation - rapidly downregulated at stress onset and upregulated under prolonged conditions - with validated targeting of HD-ZIP III genes, supporting its involvement in vascular development and drought adaptation (Wei et al. 2024). Due to their great contribution to Biological Nitrogen Fixation (BNF), legumes play an important role in effective management of fertilizers and improving soil health in sustainable agriculture (Hirel et al. 2011). They are especially sensitive to deficiencies of P, potassium (K) and sulphur (S) and toxicities of aluminum (Al) and manganese (Mn) (Kochian et al. 2004; Divito And Sadras 2014; Ángel Martín-Rodríguez et al. 2021; Lu et al. 2023; Allam et al. 2025). Both types of nutritional stress can affect nodules functioning and impose serious limitations on plant growth and development. Aluminum toxicity is the main plant growth-limiting factor, causing the rapid inhibition of root growth, inhibiting water and nutrient uptake (Ma 2007). MiRNAs responsive to Al^3+^ exposure has been described in nodules of common bean (Mendoza-Soto et al. 2015; Martín-Rodríguez et al. 2021). MiR164 and miR393 expression are highly induced and their corresponding target genes transcription factor with *N*am, *A*taf1, 2, and *C*uc2 domain (NAC1) and an auxin receptor the Transport Inhibitor Response (TIR1) are repressed in nodules subjected to a long exposure of Al^3+^. MiR164 was also described as Al^3+^-stress responsive in soybean (Zeng et al. 2012) and miR393 in *M. truncatula* (Chen et al. 2012). In *P. vulgaris*, miR1511 is involved in metal toxicity responses, targeting the *ALS3* (Aluminum Sensitive Protein 3) gene, which plays a key role in aluminum detoxification in plants (Martín-Rodríguez et al. 2021). The role of miRNAs in Al^3+^ tolerance in alfalfa is being elucidated (Aung et al. 2015; Lu et al. 2023; Allam et al. 2025). A recent study showed that downregulation of miR156 in roots under Al stress is crucial. Silencing via a short tandem target mimic (MsSTTM156) demonstrated upregulation of SPL targets, with effects that varied according to miR156 levels in the different transgenic genotypes, indicating the possibility of additional targets (Allam et al. 2025). At the molecular level, the biomass decrease in miR156OE was mainly due to altered root architecture by impaired auxin transport (IAA), mediated by the regulation of AUX1 and PIN2, which disrupts auxin gradients and inhibits root elongation (Allam et al. 2025).

The expression of several miRNAs is altered during abiotic stress responses, including temperature, salinity, drought, and nutrient availability (Singh et al. 2017). Table 1 briefly shows the miRNAs reported as regulated in response to abiotic stress, as well as their possible targets and in which species they were characterized. Understanding sRNA-guided stress regulatory networks will provide new tools for improving plant stress tolerance.

**Table 1:** Summary of studies with abiotic stress-responsive MiRNAs in legumes

miRNA	Stress type	Plant species	Main target	References
miR399	Phosphorus deficiency	*P. vulgaris*	*Phosphate2 (PHO2)*	Valdés-López et al. (2008)
miR169c	Cold stress	*G. max*	*HAP2-like transcription factor (NF-YA)*	Zhang et al. (2014)
miR156	Cold stress	*G. max*	*-*	Kuczyński et al. (2022)
miR169	Cold stress	*G. max*	*-*	Kuczyński et al. (2022)
miR5770	Cold stress	*G. max*	*-*	Kuczyński et al. (2022)
miR4415	Cold stress	*A. nanus*	*L-ascorbate oxidase (AO)*	Zhu et al. (2022)
miR396	Salt stress	*T. dicoccoides*	*Growth-regulating factor (GRF)*	Kantar et al. (2011)
				Chen et al. (2024)
miR166m	Salt stress	*G. max*	*Chloroplastic beta-amylase 1 (BAM1)*	Li et al. (2022)
miR408	Drought stress	*V. unguiculata*	*Laccase12 (LAC12)*	Mishra et al. (2022)
miR166	Drought stress	*M. truncatula*	HD-ZIP III transcription factors	Wei et al. (2024)
miR164	Al3 + stress	*P. vulgaris*	*NAC domain-containing protein 1 (NAC1)*	Mendoza-Soto et al. (2015)
miR393	Al3 + stress	*P. vulgaris*	*Transport Inhibitor Response 1 (TIR1)*	Martín-Rodríguez et al. (2021)
miR1511	Al3 + stress	*P. vulgaris*	*Aluminum Sensitive Protein 3 (ALS3)*	Martín-Rodríguez et al. (2021)
miR156	Al3 + stress	*M. truncatula*	*Auxin influx carrier AUX1 and PIN-FORMED 2 (PIN2)*	Allam et al. (2025)

### MiRNA regulation in legume defense against biotic challenges

Plants develop in complex environments, forming associations with diverse organisms that can be beneficial, such as pollinators and nitrogen-fixing rhizobia, or detrimental, including pathogens and strong competitors (Van Dam 2009; Andrews And Andrews 2017). Despite their ecological importance via rhizobia symbiosis, legume yields are limited by a broad range of pathogens (Sillero et al. 2006; Heeren et al. 2012; Hema et al. 2014; Martins et al. 2020; Costa et al. 2021; Jha et al. 2023). Plant immunity involves multilayered defenses, with miRNAs modulating pathogen responses through post-transcriptional regulation of key genes (Jones et al. 2006; Jwa And Hwang 2017; Islam et al. 2018a; Li et al. 2020). Many studies are focused on better understanding miRNA-mediated processes and describe their role within a dynamic network that mediates interactions between plants and pathogens (Luo et al. 2024a). Converging evidence further indicates that strategically manipulating sRNA circuits can inform next-generation disease control and potentially enhance crop productivity (Zhang et al. 2016; Islam et al. 2018b; Kumar et al. 2022a).

The first comprehensive characterization of known and novel miRNAs responsive to Asian soybean rust (ASR), the foliar disease caused by *Phakopsora pachyrhizi* Sydow & Sydow (Kulcheski et al. 2011). In susceptible genotypes of soybean, most miRNAs were repressed during infection, including MIR-Seq11 and MIR-Seq13, whose predicted targets encode peroxidases and oxidoreductases, respectively. These enzyme classes are typically induced following pathogen challenge, including ASR (Choi et al. 2008). Legume leaf and root diseases are associated with diverse fungal and oomycete pathogens (Al-Jaradi et al. 2018; Zhao et al. 2020a; Sharma et al. 2023; Cabral et al. 2025; Joshi et al. 2025). Few studies have shown miRNAs can possibly act as post-transcriptional regulators in legumes immune systems against fungal diseases. As an example, expression patterns of miRNAs upon infection by *Phytophthora sojae* were examined by microarray analysis in three soybean cultivars, showing 160 miRNAs responsive to *P. sojae* infection (Guo et al. 2011).

High-throughput sRNA sequencing identified miRNAs involved in peanut immunity against aflatoxin contamination by *Aspergillus flavus* (Zhao et al. 2020a). Aflatoxins are a group of mycotoxins that threaten global food safety (Gemede 2025). The results demonstrated that miR156a expression increases in resistant genotype, targeting *SPL* genes (Zhao et al. 2020a), which negatively regulates the accumulation of flavonoids with antimicrobial properties against *A. flavus* (Medina et al. 2004).*A recent study profiled miRNA expression during Aspergillus flavus infection in resistant and susceptible peanut varieties*,* identifying novel miRNAs and contrasting regulation of NBS-LRR–targeting miRNAs associated with disease resistance* (Joshi et al. 2025). The study identified 27 novel miRNAs and revealed contrasting expression patterns between varieties, notably for miR482d-3p and miR2118, which exhibited distinct temporal regulation. These miRNAs emerge as potential candidates for marker development in breeding programs, although functional validation is still required.

Among main phytopathological problems associated with legumes cultivation, nematode diseases stand out generating around 12% productivity losses annually (Davis And Mitchum 2005). Parasitic nematode species can infect legumes, but cyst and root-knot nematodes receive great attention (Dhandaydham et al. 2008; Rambani et al. 2020; Ajila et al. 2023). Soybean cyst nematode, *Heterodera glycines* L., is the primary economically limiting pest for soybean production (Niblack et al. 2006). miRNA profiling during nematode infection in soybean identified 364 known miRNAs and 21 novel miRNAs (Li et al. 2012). Furthermore, comparative analysis of 24 sRNA libraries, from two soybean cultivars with contrasting susceptibility to *H. glycines*, detected a total of 60 miRNAs that are differentially regulated in the nematode infection (Tian et al. 2017). Expression of miR399 and miR408 are highly up-regulated by *H. glycines* in the susceptible cultivar. Early-stage miRNA profiling during SCN infection in resistant and susceptible soybean cultivars revealed miR408a-3p upregulation and defense-related target genes (Lei et al. 2019). A similar expression pattern was previously reported for miR399 during *Candidatus Liberibacter asiaticus* infection in *Citrus sinensis* (Zhao et al. 2013) and for miR408 in *Triticum aestivum* in response to stripe rust, where it regulates *plantacyanin*-like protein targets (Feng et al. 2013).

Viral diseases pose a major threat to food legumes, with *Mungbean yellow mosaic India virus* (MYMIV), transmitted by whiteflies, being a major constraint on mungbean yield (Mishra et al. 2020). In *Vigna mungo* infected with MYMIV, the first miRNAs linked to antiviral immunity in legumes were identified, highlighting miR396 as part of the salicylic acid-mediated defense pathway (Kundu et al. 2017). MiR396 upregulation represses a lipoxygenase involved in jasmonic acid synthesis, which antagonizes salicylic acid signaling. Consistently, exogenous salicylic acid was previously shown to enhance MYMIV resistance in *V. mungo* (Kundu et al. 2011). Similarly, in common bean, MYMIV infection induced differential regulation of multiple miRNA families (Patwa et al. 2019). The expression of miR164c increased following viral infection (Patwa et al. 2019), and RNA Ligase-Mediated (RLM)-RACE analysis confirmed its targeting of NAC domain-containing protein 1 (Puranik et al. 2011, 2012). This transcription factor plays a central role in coordinating abiotic and biotic stress responses, functioning in an abscisic acid - independent pathway during environmental stress and through antagonistic jasmonic acid and salicylic acid signaling under biotic stress.

The interactions established between bacteria and legumes can assume both mutualistic and pathogenic characteristics, providing beneficial or detrimental effects to host plants. Plant immunological mechanisms that restrict bacterial infections have been extensively investigated, while the regulatory role of miRNAs in antibacterial immunity remains relatively underexplored (Zhang et al. 2011; Luo et al. 2014; Chow And Ng 2017). Regarding legumes, no studies to date have identified or characterized miRNAs responsive to pathogenic bacterial infections. Although legumes account for approximately 27% of global primary crop production, their yield and quality are persistently constrained by environmental challenges and pest-induced losses (Vance et al. 2000). Given their agronomic importance, substantial efforts have been devoted to developing genetic and genomic resources to support breeding programs aimed at improving resistance to biotic stresses in legume species (Díaz-Valle et al. 2019; Kankanala et al. 2019). Numerous studies have convincingly demonstrated the involvement of miRNAs in biotic stress responses, and functional analyses have shown that several plant miRNAs play critical roles in resistance to biotic stresses (Puranik et al. 2011, 2012; Zhao et al. 2020a). Table 2 summarizes miRNAs-target reported to be regulated in plants in response to diverse biotic stresses, highlighting candidates with potential relevance for pathogen resistance studies in legumes. In this context, elucidating the mechanisms that regulate the expression of biotic stress-related genes is essential for the genetic improvement of legumes (Li et al. 2011; Baldrich And Segundo 2016).

**Table 2:** Summary of studies with biotic stress-responsive MiRNAs in legumes

miRNA	Stress type	Plant species	Main target	References
MIR-Seq11	ASR infection	*G. max*	Peroxidases genes	Choi et al. (2008)
MIR-Seq13	ASR infection	*G. max*	Oxidoreductases genes	Choi et al. (2008)
miR156a	A. flavus infection	*A. hypogaea*	*SQUAMOSA promoter-binding protein-like (SPL) genes*	Zhao et al. (2020a)
miR482d-3p	A. flavus infection	*A. hypogaea*	Nucleotide-binding site–leucine-rich repeat (NBS-LRR) resistance genes	Joshi et al. (2025)
miR2118	A. flavus infection	*A. hypogaea*	Nucleotide-binding site–leucine-rich repeat (NBS-LRR) resistance genes	Joshi et al. (2025)
miR408	Ca. L. asiaticus infection	*T. aestivum*	Plantacyanin-like genes	Feng et al. (2013)
miR164c	MYMIV infection	*P. vulgaris*	*NAC domain-containing protein 1 (NAC1)*	Puranik et al. (2011), (2012)
				Patwa et al. (2019)

### MiRNA-mediated symbiosis in legume associations

In the current worldwide scenario, declining soil fertility threatens sustainable food security, while biological nitrogen fixation (BNF) plays a central role in maintaining soil nutrient balance and agricultural productivity (Roy et al. 2007). Symbiotic interactions between crop and forage legumes and soil bacteria collectively known as rhizobia represent the main nitrogen-fixing agents in agricultural systems (Ormeño-Orrillo and Martínez-Romero 2013). These associations act as natural green manure, reducing the need for chemical nitrogen fertilizers (Herridge et al. 2008; Hirel et al. 2011).

As key regulators of legume symbiosis, miRNAs play crucial roles in controlling nodulation and the progression of the symbiotic process (Boualem et al. 2008; Yan et al. 2013; Hobecker et al. 2017). Studies identified conserved and legume-specific miRNAs regulating key stages of symbiosis, including nodulation and hormone homeostasis (Hussain et al. 2018; Hoang et al. 2020).An overview of studies identifying miRNAs responsive to symbiotic interactions in legumes is provided in Table 3.

**Table 3:** Summary of studies with symbiotic-responsive MiRNAs in legumes

miRNA	Symbiotic process	Plant species	Main target	References
miR169	Nodule development	*M. truncatula*	*HAP2-like transcription factor (NF-YA)*	Combier et al. (2006
miR397	Nodule symbiotic activity	*L. japonicus*	Laccase (LAC) genes	Liang et al. (2012
miR171c	Nodule symbiotic activity	*L. japonicus*	*Nodulation Signaling Pathway 2 (NSP2)*	De Luis et al. (2012)
	Nodule symbiotic activity	*G. max*	*Nodulation Signaling Pathway 2 (NSP2)*	Hossain et al. (2019)
miR171h	Nodule primordium initiation	*M. truncatula*	*Nodulation Signaling Pathway 2 (NSP2)*	Ariel et al. (2012)
miR393j-3p	Nodule development	*G. max*	*Early Nodulin 93 (ENOD93)*	Yan et al. (2015)
miR160	Nodule primordium initiation	*G. max*	*ARF10/ARF16/ARF17*	Turner et al. (2013)

The first study addressing the role of miRNAs in legume symbiosis was conducted in *M. truncatula* (Combier et al. 2006), where the transcription factor MtHAP2-1 was found to be induced during nodule development. Silencing of *MtHAP2-1* through RNA interference caused a marked delay in nodulation and nodule growth. Previously, *HAP2* genes had been identified as targets of miR169 in *A. thaliana* (Jones-Rhoades And Bartel 2004), and miR169-mediated cleavage of *MtHAP2-1* was subsequently validated by the 5′ RACE method. Collectively, these findings indicate that miR169 contributes to the spatial regulation of gene expression during nodule differentiation.

Nodule development requires two coordinated processes: bacterial infection and nodule organogenesis (Jones et al. 2007). Flavonoids exuded by plant roots attract rhizobia in the rhizosphere (Dharmatilake And Bauer 1992), inducing the expression of bacterial nodulation genes (*nod*, *nol*, *noe*) responsible for producing and exporting the lipooligosaccharide Nod factor (Cassab 1998; Oldroyd And Downie 2004). Nod factor perception activates symbiotic signaling, leading to root hair deformation, membrane depolarization, and calcium oscillations (Gage 2004). During nodule development, both conserved and legume-specific miRNAs are differentially expressed, as identified in soybean, *M. truncatula*, and *P. vulgaris* (Subramanian et al. 2008; Lelandais-Brière et al. 2009; Yan et al. 2016; Castaingts et al. 2022). Notably, miR172 isoforms play a central role in nodule initiation, with *L. japonicus* miR172a being strongly regulated at early symbiotic stages, prior to infection thread formation (Yan et al. 2013; Holt et al. 2015).

The high-throughput sequencing analysis was performed to uncover miRNAs regulated in response to symbiosis in roots and nodules of *L. japonicus* inoculated with *Mesorhizobium loti* (De Luis et al. 2012). MiR397 and miR171c were described being involved with bacterial infection and nodule symbiotic activity. Both miRNAs expressions were investigated in wild-type plants, and in two plant mutants able to develop spontaneous nodules without rhizobial infection: *spontaneous nodule formation1* (*snf1*), encoding autoactive versions of the calcium- calmodulin kinase (Tirichine et al. 2006); and cytokinin receptor *Lotus* Histidine Kinase1 (Tirichine et al. 2007). MiR397 was significantly upregulated in nitrogen-fixing infected nodules. Its abundance in mature nodules appears to depend on both the nodule’s ability to carry out effective N₂ fixation and the presence of a compatible symbiotic bacterium (Liang et al. 2012). Moreover, it was demonstrated that the regulation of *Laccase* genes associated with copper homeostasis is regulated by miR397, suggesting an involvement of copper homeostasis in the context of nitrogen fixation. Similar profiles were observed during overexpression of miR397 in rice (Zhang et al. 2013), and in *A. thaliana*, when miR397 was repressed by N deficiency (Liang et al. 2012). MiR171c, which targets the GRAS transcription factor Nodulation Signaling Pathway 2 (NSP2) in *L. japonicus*, was induced only in inoculated *snf1/2* nodules, suggesting a role in bacterial infection rather than in nodule organogenesis (De Luis et al. 2012). Although complementation of *nsp2* mutants with an *NSP2* allele carrying silent mutations at the miR171c site restored normal nodulation, other miR171 members were shown to negatively regulate *NSP2* in *M. truncatula* and soybean (Hofferek et al. 2014; Hossain et al. 2019), indicating that miRNA-mediated silencing of NSP2 may indeed be important for nodulation.

Multiple stages of legume symbiosis, from nodule initiation to maturation and senescence, are regulated by miRNA-mediated pathways (Lelandais-Brière et al. 2009; Wang et al. 2009). A study carried out in soybean sequenced 15 sRNA libraries from different stages of nodule formation, including young, mature and senescent nodules (Yan et al. 2015). MiR393j-3p expression was significantly increased during nodule development, being negatively correlated with *Early Nodulin 93* (*ENOD93*) gene expression. Functional analysis based on miR393j-3p overexpression and *ENOD93* RNA interference lines resulted in a dramatic decrease in the number of nodules. These results suggested that miR393j-3p regulation of *ENOD93* mRNA accumulation is crucial for nodule formation (Yan et al. 2015). In 2010, Li and colleagues evaluated the effect of miR482, miR1512, and miR1515 overexpression on nodule formation in soybean roots. Overexpression of miR482 and miR1515 markedly enhanced mature nodule formation, whereas miR1512 overexpression showed no effect. These miRNAs were also expressed in roots using the Rhizobium-responsive soybean promoter Enod40, an early nodulin gene, to reduce the pleiotropic effects. The number of root nodules increased in roots expressing miR482 in a *Rhizobium*-inducible manner and a similar phenotype was found in roots expressing miR1512 (Li et al. 2010).

Moreover, miRNAs play central roles in the regulation of nodule development by modulating hormone signaling pathways. In soybean, miR160, enhancing auxin sensitivity and limiting nodule primordium formation despite normal epidermal responses to rhizobia (Turner et al. 2013). Curiously, miR160 targets the ARF10/ARF16/ARF17 repressor family demonstrating that auxin hypersensitivity controlled by miRNA can restrict nodulation (Mallory et al. 2005). Similarly, in *M. truncatula*, miR171h silences the cytokinin-regulated gene *Nodulation Signaling Pathway 2* (*NSP2*), which is essential for nodule primordium initiation, highlighting the role of miRNA-mediated repression in the precise control of cytokinin signaling during nodule formation (Ariel et al. 2012). Overall, miRNAs such as miR160 and miR171h act as key regulatory nodes that integrate auxin and cytokinin responses, ensuring the spatial and temporal fine-tuning of nodule development.

Several studies have described the mechanism of underlying cross-kingdom RNA interference between eukaryotic partners. However, although eubacteria lack classical RNAi machinery, current evidence suggests that sRNAs can also be transferred between eukaryotes and eubacteria (Panstruga And Spanu 2024). Regarding the rhizobium-legume symbiosis, it has been shown that short RNA fragments derived from the Bradyrhizobium japonicum tRNA enter soybean root cells, hijack the host AGO1-containing silencing machinery, and regulate target genes involved in establishing this association (Ren et al. 2019). Thus, this study suggests that cross-kingdom RNAi is a conserved mechanism in interacting organisms, including the plant-bacteria association. However, it remains unclear how this process is regulated, including the sorting and transport mechanisms of these sRNAs, and whether there is a specific signaling mechanism for plants to induce the generation of bacterial mobile sRNAs. In 2019, studies suggested that plant sRNAs are capable of crossing both the host plant plasma membrane and the bacterial cell envelope, where they trigger gene silencing in bacteria and contribute to antibacterial defense (Singla-Rastogi et al. 2019). However, to date, no work has demonstrated whether cross-kingdom RNAi can occur bidirectionally in the plant-rhizobium association, considering that plant sRNAs may also be capable of modulating bacterial genes.

## Biotechnological application of miRNAs in legumes

Techniques for manipulation of miRNAs and their targets have been developed aiming different biotechnological applications, such as crop improvement and miRNA-based molecular markers (Djami-Tchatchou et al. 2017; Sabzehzari And Naghavi 2019). MiRNA-based strategies can be applied in the production of cultivars more tolerant to environmental stresses, and in the improvement of agronomic traits, like grain yield, nutritional quality, and fruit and flower development (Zhou and Luo 2013).

The technique of artificial miRNAs (amiRNAs) is a gene silencing strategy based on the modification of miRNA sequences to downregulate specific targets. In this strategy, a specific miRNA sequence of interest replaces the native miRNA/miRNA* duplex within the pre-miRNA, maintaining the original secondary RNA structure while directing silencing toward the chosen gene (Sablok et al. 2011; Zhou and Luo 2013). In soybean, amiRNAs were generated to increase resistance to *Heterodera glycines*, also called soybean cyst nematode. Transgenic hairy roots of soybean were transformed with amiRNA vectors, and revealed nematode populations with reduced densities, demonstrating that the amiRNA technique can be applied in soybean resistance to soybean cyst nematode (Tian et al. 2016). In *M. truncatula*, amiRNAs were used to demonstrate the action of the *flotillin-like* gene family in infection by *Sinorhizobium meliloti*, the nitrogen-fixing symbiont of *M. truncatula.* Silencing of FLOT2 and FLOT4 by amiRNAs compromised nodule formation and function, thereby impairing nitrogen fixation. Thus, through amiRNA-based silencing, it was possible to demonstrate that *flotillin-like* gene family are required for symbiotic bacterial infection (Haney and Long 2010). Some advantages of amiRNAs over other interference RNA strategies are considered, such as target silencing is more specific than traditional RNA interference vectors; amiRNAs are more environmentally friendly compared to small-interfering RNAs; amiRNA silencing activities remain more stable over different generations (Zhou and Luo 2013; Tian et al. 2016).

Another potential biotechnological application of miRNAs in legumes involves miRNA-encoded peptides (miPEPs) derived from primary miRNA transcripts (pri-miRNAs). In *M. truncatula*, primiR171b can produce miPEPs (miPEP171b), derived from open reading frames (ORFs) present in pri-miRNAs. miPEPs perform a regulatory role, leading to the accumulation of their mature miRNA and consequent downregulation of target genes. The application of synthetic miPEP171b in *M. truncatula* generated the accumulation of miR171b, causing the reduction of lateral root density (Lauressergues et al. 2015). Furthermore, a miPEP corresponding to the miR171b homolog was identified in *L. japonicus*. Treatment with the specific miPEP171b enhanced the expression of the miR171b homolog and promoted the mycorrhizal process (Couzigou et al. 2017). In soybean, the use of synthetic miPEP172c leads to increased expression of miR172c and increases nodule number (Couzigou et al. 2016). These results demonstrate that miPEPs can be applied as potential tools to regulate agronomic traits in plants. Considering that miPEPs act by increasing the expression of their associated miRNAs, treatment with exogenous miPEPs in plants may be an alternative strategy, in which miRNA target genes can be downregulated without the use of genetic transformation (Couzigou et al. 2015). Through computational analysis, novel miPEPs were identified in Fabaceae species. In *A. hypogaea* (peanut), 14 novel miPEPs were reported (Ram et al. 2019). In soybean, *P. vulgaris*, *V. unguiculata*, and *M. truncatula*, a total of 81 putative miPEPs were identified from the miR171, miR397, miR398, miR408, and miR482 families (Araújo And Grativol 2022). The number of miPEPs in legumes and plants in general is far from being determined, and their mechanism of action still needs further studies (Lauressergues et al. 2022). However, the discoveries made so far show that miPEPs have great biotechnological potential, regarding crop improvement and as a sustainable alternative to the use of chemicals in agriculture (Ormancey et al. 2021).

Several studies have demonstrated that plant miRNAs can act in a cross-kingdom manner, regulating gene expression in animals following dietary intake, and have been detected in mammalian serum, feces, and tissues (Zhang et al. 2012; Liang et al. 2014; Yang et al. 2015). Diet-derived plant miR156 has been identified in mammalian intestines, showing that plant miRNAs influence intestine cell proliferation and intestinal tract development (Li et al. 2019). Another plant miRNA that has cross-kingdom action is miR159. MiR159 showed higher abundance in healthy donors than in patients with breast cancer, suggesting that plant-derived miR159 can inhibit cancer growth in mammals (Chin et al. 2016). Among the identified miRNAs, miR159a-3p and miR159e-3p from soybean, together with miR159a from Arabidopsis, obtained an abundance six times greater than other plant miRNAs in donor serum (Chin et al. 2016). In a study using human colonic Caco-2 cancer cells, Liu et al. (2020) demonstrated that soybean-derived sRNAs reduce cancer progression and promote apoptosis. Among the detected miRNAs, miR159a played a central role by inhibiting Transcription Factor 7, which is upregulated in colon cancer cells. In addition, miRNAs are also being considered crucial mediators of plant-microbe interactions. Probably, miRNAs, carried by extracellular vesicles, constitute a bidirectional communication channel between cross-kingdoms between the plant and its associated rhizospheric microorganisms (Middleton et al. 2020). Based on evidence that sRNAs can also be transferred between eukaryotes and bacteria (Ren et al. 2019), better understanding this plant-bacteria communication, including whether there are targets in bacteria for plant miRNAs, can lead to important contributions to the development of a tool for novel holobiont engineering. Recent studies in animals have shown that mitochondria can produce their own miRNAs (sometimes referred to as mitomiRs), which participate in cellular regulation and stress responses (Luo et al. 2024b). This raises the intriguing possibility that mitochondria-derived small RNAs in plants may similarly play regulatory roles, potentially contributing to interkingdom signaling between plants and associated bacteria.

CRISPR/Cas 9 is a formidable tool that can be used to edit both coding and non-coding genes, including miRNAs (Zhang et al. 2021). CRISPR/Cas 9 can delete a couple of nucleotides around the protospacer adjacent motif sequence (Yin et al. 2017), resulting in coding genes silencing. However, removing a couple of nucleotides will not efficiently silence miRNA genes. There have been few reports on the successfully knockout of miRNA genes using CRISPR/Cas9 (Zhou et al. 2017). Since miRNAs are potential targets for conferring abiotic stress tolerance in plants, new strategies based on CRISPR/Cas9 technology may provide accessible to silence almost all genetic locations, including miRNA. CRISPR/Cas9 systems have been successfully applied in legume plants, with significant progress achieved in recent years (Wang et al. 2017, 2020b). Considering hundreds of legume host genes are involved in the symbiotic nitrogen-fixation, root or nodule-specific promoters can be alternative choices for driving Cas9 gene expression during hairy root transformation, a rapid method to assay mutant phenotypes of nodulation-related genes (Wang et al. 2016; Shu et al. 2020).

RNA interference technology has been usually based on the use of transgenic plants expressing double-stranded RNAs against selected targets, demonstrating a great potential against invading pathogens (Joga et al. 2016). However, the use of genetically modified organisms has raised considerable scientific and public concerns. Thus, alternative approaches that avoid the use of transgenes are needed. Direct exogenous application of RNA molecules with the potential to trigger RNA interference (Dalakouras et al. 2020) is a very efficient tool in crops mainly to control plant diseases (Cagliari et al. 2019; Werner et al. 2020). Few studies describe technologies with RNA interference-based gene silencing that can be triggered in the target organism by the direct delivery of sRNAs (Gong et al. 2013). However, it is reasonable to assume that optimization of RNA production may pave the way for the exogenous application of diverse RNA molecules, including miRNAs.Although recent advances in plant genetics have revealed the central role of miRNAs as dynamic regulatory networks in mediating plant immunity (Islam et al. 2018b), further investigation is required and is expected to provide valuable insights for the development of new disease control strategies.

## Conclusion

Several recent findings have established that plants assign miRNAs as critical post-transcriptional regulators of gene expression to specific target genes in order to trigger crucial responses to the multiple environmental pressures they face during their growth stages (. Due to the great potential to regulate different pathways related to plant development and the response to environmental stimuli, researchers have explored the use of miRNAs as biotechnological tools. Notably, advances in CRISPR/Cas9 technology allow the manipulation of virtually all genomic regions, including miRNA loci, thereby accelerating legume research, particularly with respect to improving the biological nitrogen fixation (BNF) process. Furthermore, inter-kingdom communication, miRNA-based regulatory engineering, and the topical application of miRNA-encoded peptides (miPEPs) are emerging as frontier translational strategies in legume sRNA research, opening new possibilities for fine-tuning complex traits through reversible or species-specific regulatory modulation rather than extensive genomic modification. Another interesting application of studies with legume miRNAs is the potential use of these miRNAs, especially soybean miRNAs, in the treatment and prevention of cancer.

Despite these promising perspectives, significant challenges remain for the effective translation of miRNA-based technologies into breeding programs. Although many miRNAs are evolutionarily conserved, their expression patterns, regulatory networks, and functional outputs are often highly species-, tissue-, and context-dependent, limiting the direct transferability of miRNA-based strategies across crops. This regulatory divergence may lead to pleiotropic or trade-off effects on plant development and yield, particularly under variable environmental conditions. In addition, limited field validation, environmental sensitivity of miRNA expression, and regulatory constraints pose major hurdles for large-scale application. Comparative analyses across legume species, combined with functional validation under field-relevant conditions, will be essential to bridge the gap between conserved miRNA regulation and their effective application in crop improvement. A deeper understanding of the molecular mechanisms underlying miRNA-mediated post-transcriptional gene silencing can provide valuable resources for the effective application of this technology in crop improvement programs. Although much is already known about these small molecules with significant biotechnological potential, continued research is required to fully exploit their capabilities.

Figure 1: Simplified schematic model of miRNA biogenesis and action in plants. First, *MIR* genes are transcribed by RNA Polymerase II (Pol II) into the primary miRNA (pri-miRNA). This precursor having a 7 ‘methyl guanosine cap at the 5’ end and a poly-A tail at the 3 ‘end and is mainly processed by DCL1. DCL1 interacts with HYPONASTIC LEAVES 1 (HYL1) and leads to the formation of the miRNA/miRNA∗ duplex. HEN1 methylates the duplex and it can be transported to the cytoplasm by HASTY. RISC assembly can be occurring in the cytoplasm, or can also occur in the nucleus, being transported by EXPO1. The possible mechanisms of action of miRNAs are in the gray box. Additional action of miRNA is trigger the production of ta-siRNAs through siRNA biogenesis via mechanisms involving RDR6 and DCL2/4.
